# An incomplete form of anti-ganglioside antibody-positive Miller Fisher syndrome after an Epstein-Barr virus infection: A case report

**DOI:** 10.1097/MD.0000000000024451

**Published:** 2021-02-05

**Authors:** Le Chang, Jing Xiong, Yuying Xue, Jie Wang, Xurong Zhu, Xuejiao Zheng, Xiaoyu Gao, YuE Yan, Jiaduo Hao, Hehuan Zhao, Zhengli Di

**Affiliations:** a Department of Neurology, The Affiliated Xi’an Central Hospital of Xi’an Jiaotong University, Xi’an; b Department of Graduate School, Yan’an University, Yan’an, Shaanxi, China.

**Keywords:** Miller Fisher syndrome, anti-GQ1b IGg antibody, Epstein Barr virus

## Abstract

**Rationale:**

The Miller Fisher syndrome (MFS) is an acute polyradiculoneuritis regarded as an uncommon clinical variant of the Guillain-Barre syndrome (GBS). It is characterized by the clinical triad of ophthalmoplegia, ataxia, and areflexia. The diagnosis of MFS is based on clinical presentation, presence of albuminocytologic dissociation in the cerebrospinal fluid (CSF), and normal brain imaging results. The presence of anti-ganglioside antibodies (GQlb) in the serum is helpful for the diagnosis. A history of upper respiratory tract infection or diarrhea 3 days to 6 weeks before the onset of MFS is common. However, there are some patients with atypical manifestations who are difficult to diagnose. Here, we present an incomplete form of MFS where antibodies against GQ1b were detected in the serum following an Epstein Barr virus (EBV) infection.

**Patient concerns:**

A 77-year-old Chinese woman was admitted to the hospital with acute diplopia and right blepharoptosis. She had a history of mild upper respiratory tract infection 2 weeks ago. In 1 week, the symptoms rapidly progressed into bilateral ophthalmoplegia and hyporeflexia of the limbs without ataxia. CSF analysis on the third day after onset was normal, without albuminocytologic dissociation. EBV immunoglobulin G (IgG) antibodies were detected in the CSF. GQ1b and GD1b IgG antibodies were positive in the serum and negative in the CSF. No responsible lesion was found on brain imaging examination.

**Diagnoses:**

In accordance with the progressive bilateral ophthalmoplegia and hyporeflexia, the history of upper respiratory tract infection, the detection of EBV and GQ1b antibodies, and the negative brain imaging examination, the diagnosis of MFS was confirmed.

**Interventions:**

The patient was administered intravenous immunoglobulin for 5 days.

**Outcomes:**

She had a favorable outcome after treatment. At the 6-week follow-up, bilateral ocular movement limitation and tendon reflexes had recovered.

**Lessons:**

The diagnosis of MFS can be challenging, especially when encountered with incomplete symptoms and normal CSF results. Attention should be paid to the presence of anti-GQ1b IgG antibodies when the clinical manifestations are incomplete. Furthermore, EBV primary infection could be associated with MFS and considered a potential causative agent.

## 1 Introduction

Miller Fisher syndrome (MFS), first described in 1956, is an acute demyelinating polyneuropathy generally considered as an atypical variant of the Guillain-Barre syndrome (GBS). Its main clinical feature is the acute onset of the symptom triad of ophthalmoplegia, ataxia, and areflexia.^[1]^ MFS commonly presents with diplopia (78%), ataxia (48%), and both (34%). Less frequent symptoms that present with MFS include limb dysesthesia, blepharoptosis, facial, bulbar, and pupillary palsies, mild motor weakness, and micturition disturbance.^[2]^ Most patients with MFS have evidence of infection before the development of ophthalmoplegia or ataxia. In one study, 20% of the patients had *Campylobacter jejuni* infection and 8% had *Haemophilus influenzae* infection.^[3]^ The disease peaks at a median of 1 week, and improvement often starts at a median of 2 weeks. Recovery from ataxia and ophthalmoplegia usually takes 1 month and 3 months, respectively. Most patients have been reported to recover from ataxia and ophthalmoplegia 6 months after the onset of neurological symptoms.^[4]^ The diagnosis of MFS mainly depends on the 3 cardinal symptoms: ophthalmoplegia, ataxia, and areflexia. Evidence of infection before the disease and presence of albuminocytologic dissociation in the CSF also support the diagnosis of MFS. Antibodies against anti-ganglioside (GQ1b), a ganglioside component of the nerves, are associated with the disease mechanism and have been used as a diagnostic marker as well.^[5]^ Head imaging examinations are used to exclude other diseases.

## 2 Case presentation

### 2.1 Patient information

**Figure 1:**
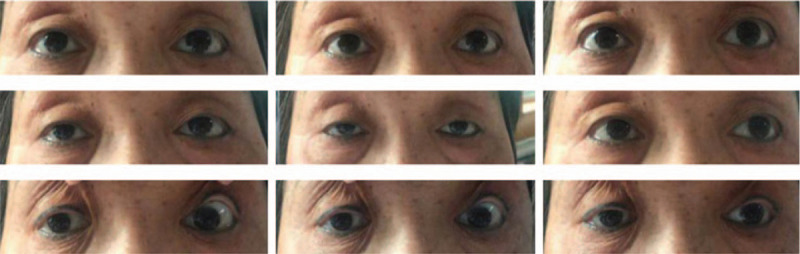
The patient had limited eye movement in all directions. This image was taken on the second day after admission.

A previously healthy 77-year-old Chinese woman was admitted to the hospital with acute diplopia and right blepharoptosis, which she had suddenly developed 1 day prior. She had experienced a mild upper respiratory tract infection about half a month before the symptoms started. She had a history of anemia 20 years before and no history of specific toxin ingestion or similar diseases. After 2 days, her symptoms progressed to bilateral blepharoptosis and restricted eye movement in all directions (Fig. 1).

### 2.2 Clinical findings

Physical examination on the first day revealed right oculomotor, trochlear, and abducens nerve paralysis and blepharoptosis. The pupil size of the bilateral eyes was not equal, and there was a sensitive pupillary reaction to light. The binocular accommodative reflex and the convergence reflex were not present. Right deviation of the tongue was observed, and the remaining cranial nerves were normal. The bilateral lower limb tendon reflexes were weakened. On the second day, bilateral blepharoptosis and oculomotor, trochlear, and abducens nerve paralysis were observed. The tendon reflex had disappeared in both lower limbs. On the ninth day, a weakened bilateral upper limb tendon reflex was observed. Limb muscle strength and tension were normal. Sensory system examinations were normal. Ataxia test, pathological reflex, and meningeal stimulation were all negative.

Blood analysis revealed anemia (low hemoglobin levels, 96 g/L) and hyponatremia (133 mmol/L). Direct anti-human globulin antibody test results were positive. Additional routine blood tests, including vitamin B12, folic acid, and thyroid function, were all within the normal range. Detection of autoantibodies was negative. Computed tomography of the bilateral orbits and the brain was normal. Neither the fatigue test nor the neostigmine test suggested myasthenia gravis. Electrophysiological examination was normal. Magnetic resonance imaging (MRI) of the brain showed the left parietal lobe, the left basal ganglia region, and a bilateral semi-oval center lacunar infarction. CSF analysis on the third day revealed normal results (proteins 301 mg/L [normal, 150–450 g/L], glucose 2.83 mmol/L [normal, 2.5–4.5 mmol/L], white cells 0/mm^3^, and red blood cells 2/mm^3^). Gram stain, acid-fast stain, and ink stain were negative. The Epstein-Barr virus (EBV) IgG was positive in CSF. Anti-ganglioside antibodies (anti-GQ1b IgG, anti-GD1b IgG, and anti-GT1b IgG) were positive in the serum and negative in the CSF.

### 2.3 Therapeutic intervention

MFS was diagnosed and intravenous immunoglobulin was administered for 5 days (20 g/d, 0.4 g/kg/d). The patient was discharged about 3 weeks after onset.

### 2.4 Follow-up and outcomes

**Figure 2:**
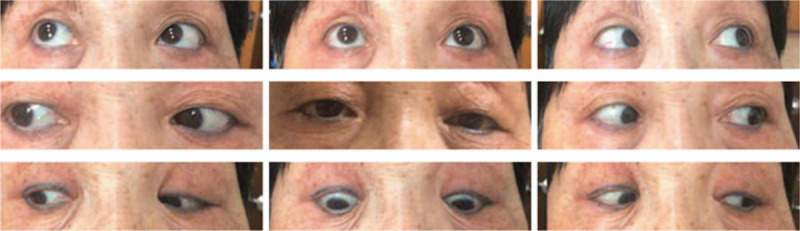
Obvious recovery of eye movement in all directions, except for the abduction of the right eye, slightly weakened. Image taken 1 and a half months after discharge.

Ocular motor function gradually improved during the follow-up period. The bilateral tendon reflexes recovered and only the abduction of the right eye was slightly weaker than normal 6 weeks after discharge (Fig. 2).

## 3 Discussion

GBS is an acute, immune-mediated inflammatory polyradiculoneuropathy of the peripheral nervous system. Characteristically, the CSF test of patients with MFS shows high protein levels with normal cell counts. When the nerve roots, which exit the spinal cord by traversing the CSF, are inflamed, proteins leak into the CSF. Since inflammation is confined to the nerve roots, there is no significant number of inflammatory cells in the CSF. Although albuminocytologic dissociation is seen in 82% to 90% of patients with GBS by the end of the second week of illness, normal protein levels may still be present during the first week.^[6]^ CSF albuminocytological dissociation is absent within the first week of symptom onset in more than half of the patients with GBS. Nerve conduction studies and CSF analyses are often inconclusive in the early stages of the disease,^[7]^ and CSF can show a normal protein level in weak patients early in presentation.^[8]^ Therefore, diagnosis and treatment we should not be delayed based on these negative results if GBS or its variants are suspected.^[7]^ Although the CSF protein levels in our patient were normal on the third day of illness (we were unable obtain new CSF protein levels due to the patient's rejection), the diagnosis of MFS could not be rejected because of the presence of ophthalmoplegia and areflexia.

Gangliosides are sialylated glycosphingolipids that are abundant in neuronal membranes and that are involved in neuronal processes including synaptogenesis, neuritogenesis, neuronal precursor migration, neuronal regeneration, and myelination.^[9]^ GM1, GD1b, GD1a, and GQ1b are important antigens, but many other gangliosides have also been identified as antibody targets.^[10]^ The localization of these target ganglioside antigens has been associated with distinct clinical characteristics. GQ1b is strongly expressed in the oculomotor, trochlear, and abducens nerves, as well as in muscle spindles in the limbs, and is associated with the MFS.^[11]^ This explains why ophthalmoplegia and ataxia are key features of MFS. In 1992, Chiba et al^[12]^ detected anti-GQ1b IgG antibodies in 6 consecutive patients with MFS, and consequently considered that it could be a useful diagnostic marker. Indeed, the presence of anti-GQ1b IgG has been confirmed in 80% to 95% of patients with MFS; therefore, it has become a useful clinical diagnostic marker of MFS.^[13]^ In fact, Spatola et al^[14]^ provided Class III evidence stating that serum GQ1b IgG accurately distinguishes MFS from other disorders. Moreover, Taams et al^[15]^ indicated that anti-GD1b antibodies may help identify patients with GBS or Miller Fisher-GBS overlap syndrome (MF-GBS). Our patient showed positive antibody results on the eighth day of the disease, and this feature helped us to identify the reason for ophthalmoplegia and to diagnose MFS.

**Figure 3:**
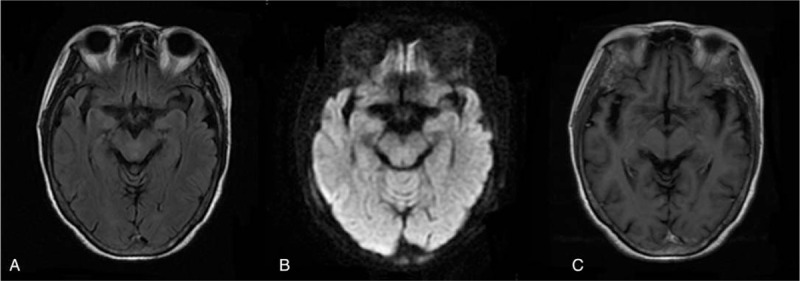
MRI of brainstem showing no abnormal signs. (A) T2-weighted flair image. (B) Diffusion-weighted image. (C) T1 contrast-enhanced image. MRI = magnetic resonance imaging.

It is also important to distinguish MFS from 2 other diseases: Bickerstaff brainstem encephalitis and Wernike encephalopathy (WE). On the one hand, Bickerstaff brainstem encephalitis has a similar pathogenesis to MFS because both are GQ1b antibody-positive autoimmune diseases caused by prodromal infection. However, in Bickerstaff brainstem encephalitis antibodies mainly enter and bind with GQ1b in the brainstem. Thus, there may be involvement of other cranial nerves, pyramidal tracts, and brainstem reticular activating systems. The clinical features of Bickerstaff brainstem encephalitis are not only ophthalmoplegia and ataxia, but also impaired consciousness and hyperreflexia. Some patients show hypodensity changes in the brainstem on brain imaging.^[16,17]^ On the other hand, WE is characterized by an acute or subacute onset of ataxia, ophthalmoplegia, and mental status changes caused by thiamine deficiency. Abnormal signs may be found in the aqueduct and third ventricle and in the medial thalamus, dorsal medulla, tectal plate, and mammillary bodies on MRI. Our patient only manifested acute progressive ophthalmoplegia and decreased tendon reflexes, and MRI of the brainstem showed no abnormal signs (Fig. 3). We diagnosed the patient with MFS and treated her with immunoglobulin. After treatment, the movement of the eyeball in all directions was restored and the tendon reflexes could be elicited, indicating great prognosis.

GBS is typically a post-infectious disorder, rapidly progressive, with a monophasic disease course shortly (<1 month) after infection, and usually without relapse. Consistent evidence suggests that GBS is an autoimmune disease caused by an immune reaction against an infectious agent that shares antigens with the nerves.^[18]^ The main pathogen to exhibit this structural similarity is *C jejuni*, which displays mimics of GM1, GD1a, GT1a, and other gangliosides on its surface lipopolysaccharide (LPS). Autoantibodies, thus, bind to glycan epitopes on peripheral nerve gangliosides, where they activate the complement and recruit macrophages, causing structural and functional disorganization of nerve conduction.^[19]^ The molecular mimicry between GQ1b and *C jejuni* LPS has been confirmed. Multivariate analysis has shown that, in GBS patients, infections caused by *C jejuni* (32%), cytomegalovirus (13%), and EBV (10%) are frequent.^[20]^ EBV infection may be associated with the autonomic variant of GBS and, rarely, with MFS. Schnorf et al^[21]^ was the first to describe a patient with MFS, high titers of anti-GQ1b antibodies, acute EBV infection, and typical symptoms. This suggests that anti-GQ1b antibodies may cross-react with EBV surface antigens. Communal et al^[22]^ reported a case of pediatric MFS complicating an EBV infection, indicting EBV as a possible causative agent of MFS. A similar molecular mechanism can be speculated in our patient: the molecular structure of the microbial LPS of EBV has molecular mimicry with GQ1b, which triggers anti-GQ1b IgG attack against autogenous neurons, which results in peripheral nerve damage. The EBV infection was likely associated with the appearance of GQ1b antibodies in our patient. However, the detailed molecular mechanism between EBV and GQ1b in MFS remains to be verified.

Only 30% of patients have negative CSF results in the first week of disease. If MFS is highly suspected based on clinical features and CSF analysis is negative in the early stages of the disease, MFS cannot be rejected. Ganglioside antibodies specifically suggest the diagnosis of MFS and they are likely to be positive in the early stage, when clinical manifestations are incomplete. In addition, primary EBV infection may be associated with MFS and could be considered a potential causative agent.

## Author contributions

**Conceptualization:** Zhengli Di.

**Writing – original draft:** Le Chang.

**Writing – review & editing:** Jing Xiong, YuYing Xue, Jie Wang, XuRong Zhu, XueJiao Zheng, Xiao Yu Gao, YuE Yan, JiaDuo Hao, HeHuan Zhao.

## References

[1] Fisher M An unusual variant of acute idiopathic polyneuritis (syndrome of ophthalmoplegia, ataxia and areflexia). N Engl J Med 1956;255:57–65. 13334797 10.1056/NEJM195607122550201

[2] Mori M Kuwabara S Fukutake T Clinical features and prognosis of Miller Fisher syndrome. Neurology 2001;56:1104–6. 11320188 10.1212/wnl.56.8.1104

[3] Koga M Gilbert M Li J Antecedent infections in Fisher syndrome: a common pathogenesis of molecular mimicry. Neurology 2005;64:1605–11. 15883324 10.1212/01.WNL.0000160399.08456.7C

[4] Hughes RA Cornblath DR Guillain-Barré syndrome. N Engl J Med 2012;366:2294–304. 22694000 10.1056/NEJMra1114525

[5] Bukhari S Taboada J A case of Miller Fisher syndrome and literature review. Cureus 2017;9:e1048. 28367386 10.7759/cureus.1048 PMC5362277

[6] Gunatilake SSC Gamlath R Wimalaratna H An unusual case of recurrent Guillain-Barré syndrome with normal cerebrospinal fluid protein levels: a case report. BMC Neurol 2016;16:161. 27596231 10.1186/s12883-016-0687-z PMC5011863

[7] Wakerley BR Uncini A Yuki N Guillain-Barre and Miller Fisher syndromes - new diagnostic classification (vol 10, p 537, 2014). Nat Rev Neurol 2014;10:537–44. 25072194 10.1038/nrneurol.2014.138

[8] Wijdicks EF Klein CJ Guillain-Barré syndrome. Mayo Clin Proc 2017;92:467–79. 28259232 10.1016/j.mayocp.2016.12.002

[9] Schengrund CL Gangliosides: glycosphingolipids essential for normal neural development and function. Trends Biochem Sci 2015;40:397–406. 25941169 10.1016/j.tibs.2015.03.007

[10] Willison HJ Anti-ganglioside antibodies in peripheral nerve pathology. Methods Mol Biol 2018;1804:173–88. 29926408 10.1007/978-1-4939-8552-4_7

[11] Paterson BJ Durrheim DN Siemieniuk RA Guillain-Barré syndrome. N Engl J Med 2012;367:973 author reply 974. 10.1056/NEJMc1208413 22931277

[12] Chiba A Kusunoki S Shimizu T Serum IgG antibody to ganglioside GQ1b is a possible marker of Miller Fisher syndrome. Ann Neurol 2010;31:677–9. 10.1002/ana.410310619 1514781

[13] Shahrizaila N Yuki N Bickerstaff brainstem encephalitis and Fisher syndrome: anti-GQ1b antibody syndrome. J Neurol Neurosurg Psychiatry 2013;84:576–83. 22984203 10.1136/jnnp-2012-302824

[14] Spatola M Du PR Schluep M Serum and CSF GQ1b antibodies in isolated ophthalmologic syndromes. Neurology 2016;86:1780–4. 26984947 10.1212/WNL.0000000000002558

[15] Taams NE Notermans NC Fokkink W-JR Clinical relevance of serum antibodies to GD1b in immune-mediated neuropathies. J Peripher Nerv Syst 2018;23:227–34. 30101437 10.1111/jns.12285

[16] Bickerstaff ER Brain-stem encephalitis; further observations on a grave syndrome with benign prognosis. Br Med J 1957;1:1384–7. 13436795 10.1136/bmj.1.5032.1384 PMC1973653

[17] Nortina S Nobuhiro Y Bickerstaff brainstem encephalitis and Fisher syndrome: anti-GQ1b antibody syndrome. J Neurol Neurosurg Psychiatry 2013;84:576–83. 22984203 10.1136/jnnp-2012-302824

[18] Nobile-Orazio E The complement story in Guillain-Barré syndrome: from pathogenesis to therapy. Lancet Neurol 2018;17:483–5. 29685816 10.1016/S1474-4422(18)30144-3

[19] Goodfellow JA Willison HJ Gangliosides and autoimmune peripheral nerve diseases. Prog Mol Biol Transl Sci 2018;156:355–82. 29747820 10.1016/bs.pmbts.2017.12.010

[20] Yuki N Infectious origins of, and molecular mimicry in, Guillain-Barré and Fisher syndromes. Lancet Infect Dis 2001;1:29–37. 11871407 10.1016/S1473-3099(01)00019-6

[21] Schnorf H Rathgeb JP Kohler A Anti-GQ1b-positive Miller Fisher syndrome in a patient with acute Epstein-Barr virus infection and negative Campylobacter serology. Eur Neurol 1998;40:177. 10026022

[22] Communal C Filleron A Baron-Joly S Paediatric Miller Fisher Syndrome complicating an Epstein-Barr virus infection. Pediatr Neurol 2016;63:73–5. 27460528 10.1016/j.pediatrneurol.2016.06.018

